# Draft Genome Sequence of an Atypical Strain of *Streptococcus pneumoniae* Isolated from a Respiratory Infection

**DOI:** 10.1128/genomeA.00822-14

**Published:** 2014-08-14

**Authors:** Sang Chul Choi, Jayme Parker, Vincent P. Richards, Katherine Ross, Bernard Jilly, Jack Chen

**Affiliations:** a Department of Biology and Wildlife, Institute of Arctic Biology, University of Alaska Fairbanks, Fairbanks, Alaska, USA; b Alaska State Public Health Virology Laboratory, Fairbanks, Alaska, USA; c Department of Population Medicine and Diagnostic Sciences, College of Veterinary Medicine, Cornell University, Ithaca, New York, USA; d Alaska State Public Health Laboratories, Anchorage, Alaska, USA

## Abstract

Next-generation sequencing was used to investigate an unknown clinical respiratory infection. This new strain of *Streptococcus pneumoniae*, ASVL_JC_0001, was isolated from a clinical specimen from a patient with bronchitis and pulmonary inflammation. The draft genome sequence, obtained with an Illumina MiSeq sequencing system, consists of 83 large contigs, a total of 2,092,532 bp long, and has a GC content of 40.3%.

## GENOME ANNOUNCEMENT

*Streptococcus pneumoniae* is a Gram-positive, α-hemolytic, aerobic bacterium belonging to the *Streptococcaceae* family (1). *S. pneumoniae* is the most frequently isolated respiratory pathogen in community-acquired pneumonia (2–5). The infections due to *S. pneumoniae* include otitis media (6, 7), sinusitis, peritonitis, and rare cases of endocarditis (8). *S. pneumoniae* colonizes the upper respiratory tract, especially in children, with an estimate of one infection for every child up to the age of 6 years in the United States (1, 9, 10). The most closely related species on the basis of 16S rRNA sequences are *Streptococcus oralis* and *Streptococcus mitis*, which share over 99% sequence identity with *S. pneumoniae*, although genomic similarities for the entire chromosome are estimated to be <60% (11–13).

We isolated a new strain of *Streptococcus pneumoniae* (ASVL_JC_0001) from a clinical specimen from a patient with bronchitis and pulmonary inflammation. This strain showed atypical features of *S. pneumoniae*, including alpha-hemolytic colonies, bile solubility, and resistance to optochin (13–15). Antimicrobial susceptibility testing showed that this strain was sensitive to cefotaxime, chloramphenicol, oxacillin, penicillin, tetracycline, and vancomycin, but it was resistant to erythromycin and ethyl hydrocupreine and partially resistant to sulfamethoxazole trimethoprim (16–21). PCR assay of housekeeping genes for *S. pneumoniae* indicated that this variant harbors genes encoding the virulence factors pneumolysin (*ply*) and the major autolysin (*lytA*), both of which are normally associated with pneumococci (22–26).

A pure culture was obtained by growing the isolate on blood agar plates at 37°C with 5% CO_2_. To perform genomic sequencing, bacterial growth from the overnight culture was suspended in 1 mL of 0.85% saline. The bacterial cells were pelleted by centrifugation at 2,300 × *g* for 15 min, and the genomic DNA was extracted and purified using a Qiagen DNeasy blood and tissue kit (Qiagen, Inc., Valencia, CA). Purified genomic DNA was then used to prepare a sequencing library using a Nextera DNA sample preparation kit (Illumina, San Diego, CA). Genome sequencing was performed on an Illumina MiSeq version 2 system, which generated 44 to 50 million paired ends (2 × 250 bp), for a total of 7.5 to 8.5 Gbp.

We assembled *de novo* genome sequences using SPAdes version 2.5.1 (27). The bacterial genome assembly consists of 83 contigs, ranging in size from 203 bp to 323,279 bp (median, 997 bp; mean, 25,210 bp). The total base length is 2,092,532 bp, with a GC content of 40.3%. The proportions of each of the DNA characters are 22.9%, 25.7%, 28.6%, and 22.8%, with 56× coverage. We annotated the genome assembly by using the NCBI Prokaryotic Genomes Automatic Annotation Pipeline (28). Among a total of 83 contigs, 69 contigs harbor annotations of genes, coding sequences (CDS), and RNAs. We found 2,158 putative genes and 2,051 CDS. We also found 5 rRNAs, 44 tRNAs, and 1 noncoding RNA (ncRNA). The maximum number of proteins annotated was 321, and 15 contigs did not contain annotated proteins. One of the contigs has only tRNAs and rRNAs.

### Nucleotide sequence accession numbers.

The draft genome sequence of *S. pneumoniae* strain ASVL_JC_0001 has been deposited at DDBJ/EMBL/GenBank under the accession number JJMK00000000. The version described in this paper is version JJMK01000000.
